# Co-administration of H-ferritin-doxorubicin and Trastuzumab in neoadjuvant setting improves efficacy and prevents cardiotoxicity in HER2 + murine breast cancer model

**DOI:** 10.1038/s41598-020-68205-w

**Published:** 2020-07-10

**Authors:** F. Andreata, A. Bonizzi, M. Sevieri, M. Truffi, M. Monieri, L. Sitia, F. Silva, L. Sorrentino, R. Allevi, P. Zerbi, B. Marchini, E. Longhi, R. Ottria, S. Casati, R. Vanna, C. Morasso, M. Bellini, D. Prosperi, F. Corsi, S. Mazzucchelli

**Affiliations:** 1 Nanomedicine Laboratory, Department of Biomedical and Clinical Sciences “Luigi Sacco”, Università Di Milano, Milan, Italy; 2 Breast Unit, Istituti Clinici Scientifici Maugeri IRCCS, Pavia, Italy; 3 Pathology Unit, Department of Biomedical and Clinical Sciences “Luigi Sacco”, Università Di Milano, Milan, Italy; 4 Medical Chemistry, Department of Biomedical and Clinical Sciences “Luigi Sacco”, Università Di Milano, Milan, Italy; 5 Nanomedicine and Molecular Imaging Laboratory, Istituti Clinici Scientifici Maugeri IRCCS, Pavia, Italy; 6 NanoBioLab, Department of Biotechnology and Biosciences, Università Di Milano-Bicocca, Milan, Italy; 7 Department of Biomedical, Surgical and Dental Sciences, Sezione Di Tossicologia Forense, Università Di Milano, Milan, Italy; 8 Present Address: Division of Immunology, Transplantation and Infectious Diseases, IRCCS San Raffaele Scientific Institute and Vita-Salute San Raffaele University, 20132 Milan, Italy; 9 Present Address: Tumor Biology and Vascular Targeting Unit, Division of Experimental Oncology, IRCCS San Raffaele Scientific Institute and Vita-Salute San Raffaele University, 20132 Milan, Italy

**Keywords:** Breast cancer, Breast cancer

## Abstract

Neoadjuvant chemotherapy has been established as the standard of care for HER2-positive breast cancer since it allows cancer down-staging, up to pathological complete response. The standard of care in the neoadjuvant setting for HER2-positive breast cancer is a combination of highly cytotoxic drugs such as anthracyclines and the anti-HER2 monoclonal antibody. Despite this cocktail allows a pathological complete response in up to 50%, their co-administration is strongly limited by intrinsic cardiotoxicity. Therefore, only a sequential administration of anthracyclines and the anti-HER2 treatment is allowed. Here, we propose the anthracycline formulation in H-Ferritin nanocages as promising candidate to solve this unmet clinical need, thanks to its capability to increase anthracyclines efficacy while reducing their cardiotoxicity. Treating a murine model of HER2-positive breast cancer with co-administration of Trastuzumab and H-Ferritin anthracycline nanoformulation, we demonstrate an improved tumor penetration of drugs, leading to increased anticancer efficacy and reduced of cardiotoxicity.

## Introduction

In recent years, neoadjuvant chemotherapy (NACT) has been established as the standard of care for HER2-positive breast cancer (BC)^1^. Indeed, treating patients by NACT before surgery allows cancer down-staging, up to pathological complete response (pCR) both in primary tumor and in the axilla. The main advantage is to make operable inoperable lesions, or to convert the indication for total mastectomy and complete axillary dissection into more conservative surgical approaches^2^. Moreover, pCR is highly predictive of favourable long-term oncologic outcomes^3^. Thus, obtaining pCR is currently the main goal of NACT. The mainstay of NACT for HER2-positive BC is the anti-HER2 monoclonal antibody trastuzumab (TZ), since its introduction in clinical practice has profoundly changed the natural history of this aggressive subtype of BC^4,5^. However, TZ as a single therapy has a scarce antitumor activity and needs to be associated with a highly cytotoxic drug such as an anthracycline^6^. Combination of anthracyclines with TZ has dramatically increased the pCR rate up to 50%, thus establishing itself as the standard NACT regimen in HER2-positive BC^2^. However, the great efficacy of these two drugs is strongly limited by their intrinsic cardiotoxicity, both related with acute adverse effects and chronic development of left ventricular systolic dysfunction^7^. Therefore, administration of anthracyclines and TZ is sequential in current clinical practice. But this higher efficacy is counterbalanced by overwhelming cardiotoxicity, leading to heart failure in up to 27% of patients^8^. Administering NACT with concurrent use of anthracyclines and TZ is therefore a clinical unmet need. Nanomedicine holds a great potential in responding to currently unsolved issues, providing smart drug delivery systems which are specifically targeted toward cancer cells, thus limiting off-target toxicity. The novel anthracycline formulation exploiting H-Ferritin (HFn)-nanocages has proven to be the optimal candidate for clinical translation in BC therapy, thanks to its capability to increase doxorubicin (DOX) anticancer effect while reducing its cardiotoxicity^9^. The aim of the present study was to assess the efficacy and the cardiotoxicity of a combined NACT with nanoformulated DOX (HFn-DOX) and TZ in a murine model of HER2-positive BC.

## Results

### The combination of HFn-DOX with TZ displays increased antitumor potential

**Figure 1:**
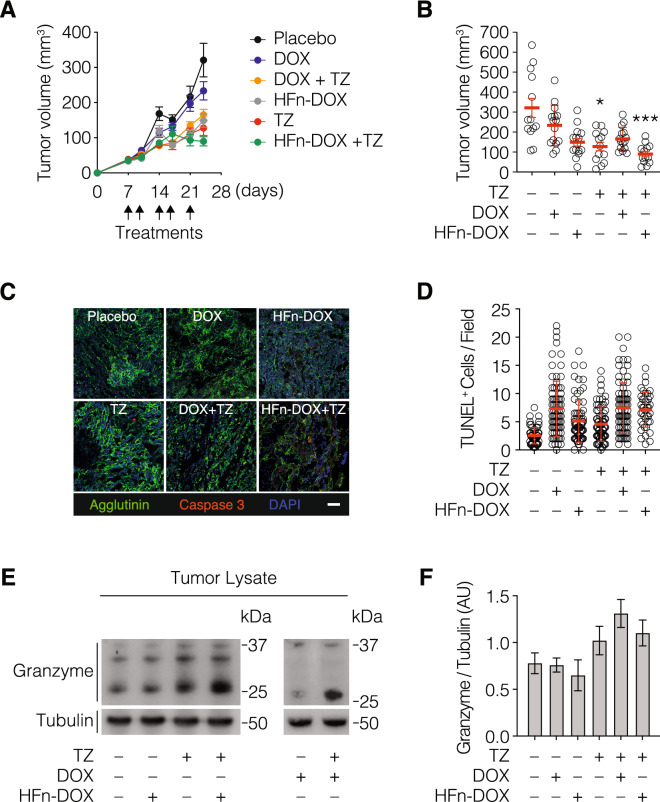
In vivo efficacy of the combined treatment with TZ and HFn-DOX. (**A**) Tumor progression in D2F2/E2 tumor bearing BALB/c mice (n = 15/group) treated with placebo, HFn-DOX or DOX (1 mg/Kg of DOX, i.v.), TZ (5 mg/Kg, i. p.) and with the combination of them (HFn-DOX + TZ or DOX + TZ). (**B**) Tumor volume measured at day 24. Plot reported individual values ± s.e. Statistical significance vs placebo *P < 0.05; ***P < 0.005. (**C**) Immunofluorescence with cleaved caspase 3 antibody (red) on histological slides of OCT embedded tumors excised at day 24. Magnification 40 ×. Nuclei have been labelled with DAPI (blue) and cellular membranes with Alexa Fluor488-Wheat Germ Agglutinin (green). Scale bar = 30 µm. (**D**) Quantification of apoptosis in tumor tissue upon treatment (n = 8/group). Reported values are the mean of apoptotic cells number/field/sample ± s.e. The count was performed on 10 fields/sample. Statistical significance vs. placebo ***P < 0.0005; ****P < 0.0001; ^#^P < 0.05, ^##^P < 0.01, ^###^P < 0.005. (**E**) Quantification of western blot analysis performed on tumors excised at the end of treatment to quantify granzyme release. Granzyme was normalized on tubulin using ImageJ Software. Reported values are the mean of 3 samples ± s.e. (**F**) Representative results of western blots performed to quantify Granzyme, constituted by cropped western blots revealed with anti-Granzyme B antibody and anti-α tubulin, respectively.

To assess the feasibility of the combined administration of the anthracycline Doxorubicin and the anti-HER2 targeted therapy with TZ, we exploited the “cardiosafe” formulation of DOX named HFn-DOX. BALB/c female mice bearing D2F2/E2 tumor, which is a murine BC expressing human HER2 receptor and hence responsive to TZ, were randomly divided into six experimental groups at day 7 and treated with placebo, HFn-DOX or free DOX, TZ or with the combination of HFn-DOX + TZ or DOX + TZ. The treatment was repeated twice a week for 2 weeks and half. Growth profiles reported in Fig. 1A evidences that both the combination of and the single treatments with nanoformulated DOX (i.e. TZ and HFn-DOX) were effective in reducing tumor growth in comparison to the group treated with placebo. Despite this, in the groups treated with TZ or HFn-DOX as single agent, we could only slow down tumor growth, while in the group treated with the combination of TZ and HFn-DOX we could notice tumor regression. DOX displays lower efficacy that nanoformulated HFn-DOX, as expected from previous evidences in Triple-Negative Breast Cancer murine model^9^. Therefore, the growth profile of this group is quite similar to placebo. Moreover, also in group treated with the combination of DOX and TZ, the efficacy in comparison to placebo seems to be similar to that observed in groups treated with TZ or with HFn-DOX as single agents. These observations were confirmed by tumor size measurements performed after tumor dissection (Fig. 1B). Tumor size reduction is statistically significant in comparison to placebo only in groups treated with TZ as single agent or in combination with HFn-DOX, despite tumor sizes observed in mice treated with TZ alone were larger than those measured in the group treated with the combination of HFn-DOX and TZ, suggesting an additive effect due to the combination of these drugs. To characterize in depth the anticancer effects of TZ and HFn-DOX combination, we evaluated the capability to induce apoptosis by cleaved caspase 3 staining (Fig. 1C), tunel assay (Fig. 1D) and Granzyme B release upon Antibody Dependent Cell-mediated Cytotoxicity (ADCC), (Fig. 1E, F). As expected, the labelling with cleaved caspase 3 antibody confirms results of tumor size, since the red fluorescence signal of cleaved caspase 3 is clearly detectable only in samples from HFn-DOX + TZ group. An increase in the number of apoptotic nuclei has been detected in tumor samples from drug-treated mice in comparison to ones from mice of the placebo group. Single treatment with HFn-DOX was more effective than DOX in induce nuclei fragmentation, both as single treatment, both in combination with TZ. Although differences between the group treated with TZ alone and HFn-DOX or DOX alone have not been evidenced, mice treated with their combination displayed a higher amount of apoptosis in comparison to ones treated with a single drug, suggesting a synergic effect of TZ-anthracycline combination. Since the engagement of Fc gamma receptor-expressing leukocytes—especially natural killer cells (NK)—with TZ has been shown to potently induce ADCC^10^, we evaluated by western blot the presence of Granzyme B in tumor lysates as readout (Fig. 1E, F and Supplementary Fig. 5). As expected, the amount of this cytotoxic protein was much more abundant in groups treated with TZ. The higher amount of Granzyme B was actually due to cellular activation and not to an enhanced intratumor leukocyte recruitment, given that the percentage of NK, T cells and NK-T cells did not vary among cured TZ and HFn-DOX + TZ groups (Supplementary Fig. 1). Otherwise in DOX + TZ group, the increase in Granzyme B is coupled with an increase in NK population suggesting an enhancement in intratumor leukocyte engagement. Importantly, we found that ADCC occurred in similar extent in samples from mice treated with TZ as single agent or in combination with HFn-DOX or DOX, indicating that the presence of Doxorubicin did not interfere with the effector functions of tumor-infiltrating leukocytes.

### HFn-DOX protects against TZ-induced mitochondrial cardiotoxicity

**Figure 2:**
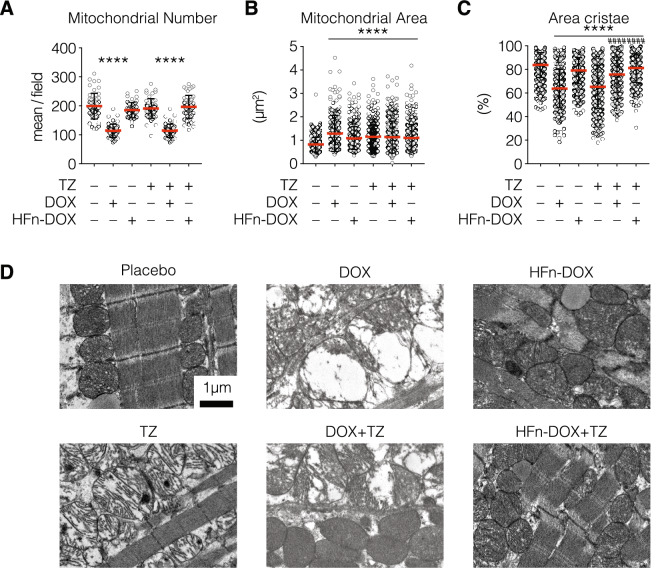
Analysis of HFn-DOX and TZ mitochondrial cardiotoxicity. Hearts from treated mice were fixed with glutaraldehyde and embedded in epoxy-resin. At least 10 TEM images of ultrathin heart sections have been acquired at 4,200 and 11,500 magnifications for each experimental group. Quantification of mitochondria area and area occupied by cristae were performed on at least 10 images/group, measuring at least 100 mitochondria/sample, while count of mitochondria number were performed on at least 10 images/group. Values represent the mean mitochondria number ± s.e. (**A**), the mean mitochondrial area ± s.e. (**B**) and the percentage of mitochondrial area occupied by cristae ± s.e. (**C**). Statistical significance vs. Placebo ****P < 0.0001; vs TZ ^####^P < 0.0001 (Kruskal-Wallis test). (**D**) Representative images of mitochondria from hearts excised at day 24 (n = 3/group) from treated mice acquired at 11,500 magnifications evidenced mitochondria morphological alterations.

Once documented the improved anticancer activity of the TZ and HFn-DOX combination in comparison to the single treatments, we assessed their toxicity in off-target organs with particular regard to the heart. Indeed, cardiotoxicity represents the main side-effect associated with DOX administration and it is also a drawback often associated to TZ treatment, since HER2 overexpression plays a pivotal role also in cardiomyocytes growth, survival and inhibition of apoptosis^11,12^. Both neoadjuvant and adjuvant clinical trials have challenged the notion that TZ should not be administered with DOX^13^, despite pre-clinical and clinical studies demonstrated that their coadministration results in the significant enhancement of antitumor efficacy^14–16^. To verify whether HFn-DOX could make feasible the therapeutic combination of TZ and DOX in the management of HER2^+^ BC, we analyzed heart tissue of mice treated with placebo, TZ, DOX, HFn-DOX, and with the combination of HFn-DOX + TZ and DOX + TZ in order to find typical morphological mitochondrial alteration due to cardiotoxicity. Detailed ultrastructural analysis of cardiac tissue revealed alterations in mitochondria homeostasis in groups treated with DOX both as single agent or as combination with TZ, while in groups treated with TZ, HFn-DOX and HFn-DOX + TZ the amount of mitochondria is the same to that observed in placebo group. Besides, an increase in mitochondria area coupled with cristae depletion has been detected in cured arms (Fig. 2B,C). More severe clinical picture were detected in groups treated with TZ or DOX as single agents, suggesting their crucial role in induce cardiotoxicity. The absence of massive alterations in cardiac mitochondria from mice treated with HFn-DOX alone was not surprising, since we have already demonstrated in a previous work that the nanoformulation in HFn prevents DOX cardiotoxicity in mice bearing 4T1 tumors^9^. However, it is surprising that the TZ-induced cardiotoxicity was almost completely reverted in samples from mice treated with the combination of TZ and HFn-DOX, despite the amount of DOX in heart was the same to that measured in HFn-DOX treated mice (Supplementary Fig. 2). Also, DOX cardiotoxicity is not surprising and it is clearly attributable to the increased accumulation of DOXol. Indeed, this toxic metabolite of DOX is undetectable in samples from mice treated with HFn-DOX as single agent or in combination with TZ, but it has been revealed in heart tissue homogenates of DOX and DOX + TZ treatment’s groups. Other signs of off-target toxicity were not detected as demonstrated by histopathological examination of off- target organs and by functional liver and kidneys functional enzymatic assays (Supplementary Fig. 3 and Supplementary Tables 1 and 2).

### HFn-DOX coadministration reduces TZ accumulation in heart and improves its tumor penetration

**Figure 3:**
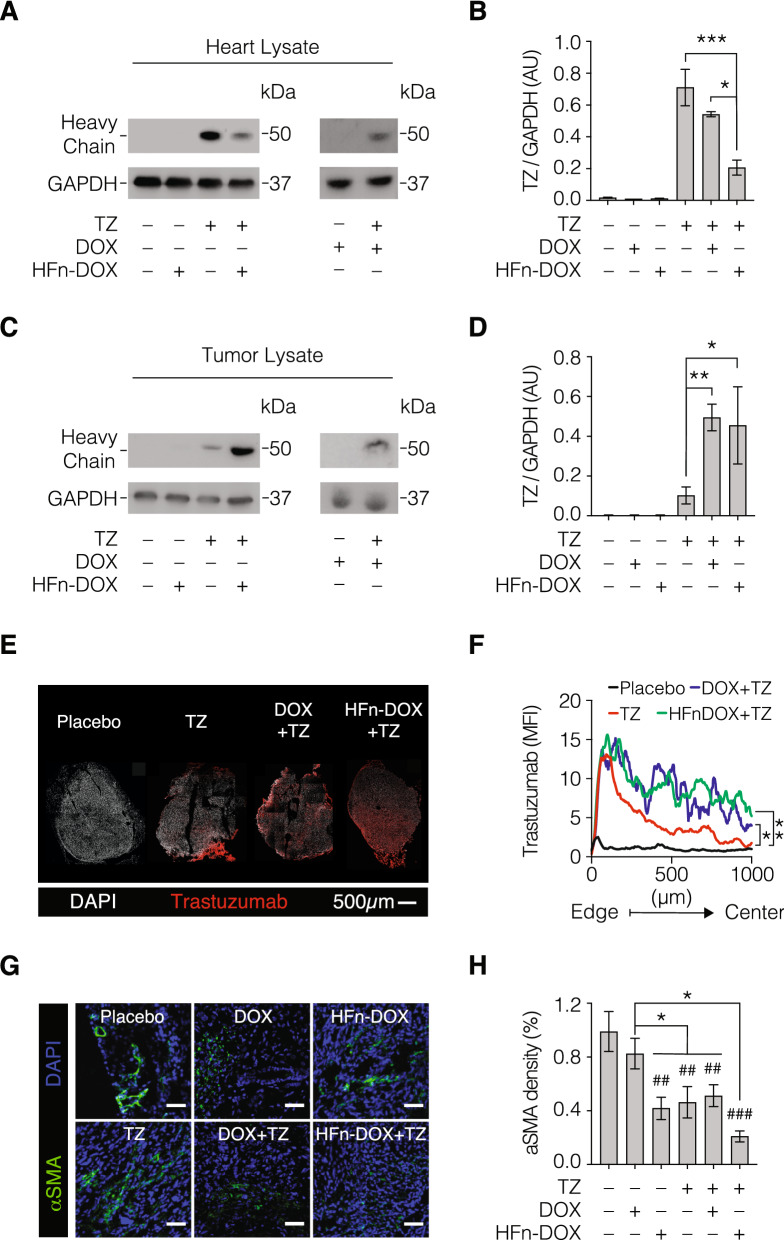
TZ quantification in heart and tumor tissue. Western blot analysis of hearts (panels A and B) and tumor (panels C and D) excised at the end of treatment to quantify TZ. (**A**,**C**) Representative results of western blots performed to quantify TZ, constituted by cropped western blots revealed with anti-human IgG HRP antibody and anti-GAPDH, respectively. (**B**,**D**) TZ quantification in heart and tumor, respectively. TZ was normalized on GAPDH using ImageJ Software. Reported values are the mean of 3 samples ± s.e. Statistical significance vs. TZ, *P < 0.05, **P < 0.01, ***p < 0.005. (**E**) Representative images of confocal microscopy analysis of TZ penetration in tumor from mice treated with placebo,TZ alone or in combination with HFn-DOX or DOX. DAPI signal is white, while TZ is labelled in red. Scale bar = 500 μm. (**F**) Analysis of spatial distribution of TZ fluorescence signal. Quantification of mean fluorescence intensity (MFI) of anti-Trastuzumab staining along the tumor axis from the edge to the geometric center (mean ± s.e [s.e. bars in grey color]; n = 3 tumor mosaics/staining; statistical comparisons between measurement points are shown). The black line represents the calculated average background of stained tumor cryosections from mice treated without trastuzumab (n = 3). Statistical significance vs. TZ, *P < 0.05, **P < 0.01. (**G**) Representative images of α-SMA labelling in frozen cryosections of tumors from treated mice (n = 3/group) to assess the amount of Cancer Associated Fibrolasts (CAF). (**H)** Quantification of the fluorescence signal of α-SMA labelling. Reported values are the mean of 3 samples ± s.e. Statistical significance vs. placebo ^##^P < 0.01, ^###^P < 0.005; vs DOX *P < 0.05.

To better investigate the rationale of this unexpected results, we tried to quantify the amount of TZ in heart lysates by western blot. Results reported in Fig. 3A,B and Supplementary Fig. 6 evidenced that TZ was significantly higher in heart from mice treated with TZ alone, despite the TZ dosage was the same administered in group treated with DOX + TZ and HFn-DOX + TZ. This result suggests that the co-treatments with HFn-DOX or DOX alter the TZ biodistribution and off-targeting accumulation. One possible explanation is that the killing of cancer cells resulting from HFn-DOX or DOX treatment, is able to modify TZ penetration in cancer. To assess this hypothesis, we quantified the amount of TZ in tumor lysates by western blot. Results reported in Fig. 3C,D and Supplementary Fig. 7 clearly evidenced that TZ was higher in samples from mice treated with HFn-DOX + TZ or DOX + TZ, while it was very low in samples from mice treated only with TZ. This first evidence strongly supports our hypothesis, revealing that more TZ reached the tumor, less TZ accumulated in off-target organs. To finally assess if the higher accumulation in cancer was really due to improved TZ penetration, we performed a study of TZ localization. We obtained confocal microscopy images of the whole tumor section by mosaic acquisition, as reported in Fig. 3E. Image analysis of reconstituted tumor section confirmed the improvement in TZ tumor penetration following the combined treatment with HFn-DOX or DOX, evidencing the multiple positive effects due to a drug formulation with better targeted delivery (Fig. 3F). To evaluate the hypothesis that the better drug penetration of TZ is due to the HFn-DOX or DOX effect against tumor stroma, we assessed the amount of Cancer Associated Fibroblasts (CAF) in tumor tissue evaluating the expression of α-Smooth Muscle Actin (α-SMA). Indeed, CAFs have the major role to create a barrier that prevent drug penetration in tumor^17^. The analysis of α-SMA fluorescence signal in tumor cryosections evidenced that drug treatments reduced α-SMA expression, suggesting a negative effect on CAF for both single treatments and the combination (Fig. 3G,H). However, α-SMA biomarker is expressed also in tumor vessel and its reduced expression could also be ascribed to an alteration of tumor angiogenesis due to different treatments. To elucidate this point, immunohistochemistry of CD31 antigen was performed (Supplementary Fig. 4) demonstrating that the amount of vessels in tumor tissue was not significantly affected by treatments. Therefore, the decrease in α-SMA expression observed in tumor cryosections from treated mice is ascribable to CAF reduction. Moreover, the effect observed in mice treated with the TZ and HFn-DOX combination is the highest, despite DOX + TZ, TZ, DOX and HFn-DOX employed as single agents already decreased α-SMA labelling in comparison to samples from placebo group.

## Discussion

The need to reduce DOX and TZ cardiotoxicity in HER2^+^ BC patients explains the reason of their sequential administration^18^. However, it results in elongation of treatment time and potentially in reduction of efficacy compared to concurrent administration. In the neoadjuvant setting, concurrent administration of DOX and TZ, not only would probably allow a higher pCR rate^19^, but it also would let to immediately assess response to NACT. In the present study, the concurrent administration of anthracyclines and TZ was allowed by the exploitation of a DOX nanoformulation with proved cardiosafety^9^. HFn-DOX has been widely studied for the nanometronomic treatment of highly aggressive BC, displaying good anticancer activity and poor off-target accumulation with fast liver metabolism and rapid clearance^9^. This DOX nanoformulation exploits the tumor targeting specificity of HFn nanocage, as a result of the specific binding between HFn and the transferrin receptor 1 (TfR1), which is overexpressed in cancers^9,20^. Moreover, HFn-DOX takes also advantage from its self-triggered mechanism of DOX release in the nucleus, that is a consequence of its natural nuclear homing^20^. On one hand, the nanovector’s features allow to improve DOX anticancer activity, since the tumor targeting specificity and nuclear homing of HFn have been demonstrated to give a crucial contribution in improve HFn-DOX antitumor activity. On the other hand, they play a role in limit accumulation in off-target organs and hence cardiotoxicity^9^. The heart is a preferential site of toxicity for BC therapies, since TZ recognizes the HER3 receptor expressed on cardiomyocites and affects their pro-survival pathway^21^, while anthracyclines, through inhibition of topoisomerase 2β, activate cell death pathways and inhibition of mitochondrial biogenesis^21^. Therefore, correctly addressing the relevant cytotoxic capability of DOX into cancer cells by a properly designed drug delivery system would answer to a clinical unmet need. The present study showed that the main consequence of HFn-DOX active targeting toward BC cells results in the lacking of cardiotoxicity^9^. In this scenario, the safe profile exhibited by HFn-DOX treatment, even after concurrent administration of TZ, represents a promising result.

As demonstrated in Fig. 3, the reason of this result is attributable to variations of systemic drug accumulation in target and off-target organs upon different therapeutic treatments. Surprisingly, the lower cardiotoxicity of concurrent administration of TZ and HFn-DOX is not only related to the lower internalization of DOX into cardiomiocytes, but also to lower accumulation of TZ^22^. Indeed, the TZ quantification in heart homogenates evidenced that the absence of cardiotoxic alterations observed in morphological analysis of cardiac mitochondria is coupled with a low accumulation of TZ in cardiac tissue from mice co-treated with TZ and HFn-DOX. A possible explanation for this unexpected result might be suggested by the role of CAF in drug delivery and interaction with cancer cells. Indeed, the concurrent administration of TZ and HFn-DOX demonstrated the highest cytotoxic effect on CAF, assessed by expression of α-SMA. Lacking the protective role of CAF toward cancer cells, a greater penetration of TZ was noted in tumor cells when HFn-DOX was co-administered, despite the same amount of TZ was used in experimental groups. Therefore, lower accumulation of TZ was found in heart homogenates, further explaining the reduction of cardiotoxicity. This increased penetration seems to be due to a cytotoxic effect on CAFs. Indeed, confocal microscopy imaging performed to enumerate CAFs in tumors upon different treatment evidenced that both TZ accumulation and drugs anticancer efficacy is inversely related to CAFs amount in tumor, suggesting that HFn-DOX treatment modifies tumor microenvironment (TME), with important consequences both in treatment’s efficacy, both in toxicity.

In other words, by modulating the action of DOX toward BC cells and TME thanks to nanoformulation, TZ is also easily addressed at tumor sites, lowering accumulation in off-target organs. Beyond the fact that TME acts as a barrier to prevent drug penetration, also immune-modulating effects of TME might be affected by HFn-DOX, reducing the inhibition of cytotoxic T cells and natural killer T cells by CAF and enhancing the action of immune-related treatments such as TZ^23^.

A cardiosafe anthracycline is still a mirage for oncologists. Several formulations of anthracyclines have been tested, including epirubicin, but none of them has demonstrated a significant reduction of heart damage compared to DOX^24^. A point of controversy is that clinically relevant heart failure is relatively infrequent, accounting for about 5% of patients treated with a cumulative dose of anthracyclines equal to 400 mg/m^2^^25^. But it represents only the “tip of the iceberg” of cardiotoxicity, since accounting also for asymptomatic left ventricular dysfunction, 18% of patients show cardiotoxicity after a cumulative dose of 350 mg/m^2^, and 65% after a total dose of 550 mg/m^2^^25^. Actually it is not clear which proportion of patients with asymptomatic ventricular dysfunction will develop clinically relevant heart failure, but the magnitude of this therapeutic concern is real^26,27^. Clinical translation of concurrent therapy with TZ and HFn-DOX might address this major issue.

## Methods

### HFn-DOX production

HFn nanocages were produced and purified as previously reported^20^ DOX loading procedure has been performed using the pH-dependent disassembling/reassembling procedure already reported^20^ and characterized by absorbance, fluorescence and dynamic light scattering analysis.

### Cell cultures

D2F2/E2 cell line was used to establish the murine model of orthotopic HER2 positive-BC cells. It stably expresses human HER2 receptor and has been obtained by Prof. Wei Zen-Wei (Wayne University) in 2016 from by MTA. D2F2/E2 cells were cultured in DMEM high glucose containing Sodium Pyruvate (1%), fetal bovine serum (10%), MEM non-essential aminoacids (1%), L-glutamine (2 mM), penicillin (50 UI mL^−1^), streptomycin (50 mg mL^−1^) and G418 (0.8 mg mL^−1^) at temperature of 37 °C, in 5% CO_2_. Cells were passaged using trypsin/EDTA and amplified for 2–4 weeks, before tumor implant. All cell culture reagents were purchased by Euroclone (Italy).

### Production of orthotopic tumor model

BALB/c mice (female, 8-week old) provided by Charles River Laboratories (Calco, Italy), were maintained in the animal facility, housed in single cages without food limitation and monitored daily^9^. Anestesia has been performed by inhalation of 2.5% isofluorane. Animals were handled in accordance with an experimental protocol approved by the Italian Ministry of Health. Sixty BALB/c mice were subjected to abdominal trichotomy two day before the injection of tumor cells. D2F2/E2 cells (1 × 10^6^ cells /animal) were suspended in cold serum-free DMEM high glucose medium and ortotopically injected into mammary gland. Mice wellness were monitored daily, and tumors were measured every two days. About 5 days after the implant, a small primary nodule (about 0.3 cm × 0.3 cm) is visible.

### Study design

The hypothesis to test was that DOX nanoformulation in H-Ferritin nanocages would allow its contemporary administration with TZ exhibiting higher antitumor efficacy without displaying cardiotoxicity. HFn-DOX or DOX were administered intravenously (i.v.) in tail vein at the DOX dosage of 1 mg kg^−1^, which is about 1/10 of the average amount of DOX administered in Dose-Dense regimen^28^, while TZ was intraperitoneally (i.p.) injected at the dosage of 5 mg kg^−1^. Six groups of treatment were performed (N = 15 mice/group): placebo, HFn-DOX alone, DOX alone, TZ alone, HFn-DOX + TZ and DOX + TZ. Mice have been treated 5 times, with twice treatments a week. The number of animals for each experimental group was calculated with a statistical power of at least 80 ± 5%. The experimental endpoint was set up at 24 days after cells injection to assess in vivo antitumor activity and cardiotoxicity due to different treatments. This time point was chosen to work in compliance with the European and Italian laws about welfare of research animal and in the meantime to discriminate differences in antitumor efficacy and/or cardiotoxicity due to different treatments. Before starting treatments, mice were randomly divided in experimental groups by primary tumor size. Rodents were monitored measuring tumor size progression in blind by the same operator using the caliper. Tumor volume has been determined using the following equation: Tumor volume (mm^3^) = lenght × (width)^2^/2.

Resected tissues from animals euthanized at day 24 were analyzed to evaluate the effects of different treatments on tumor growth, tumor angiogenesis and also cardiotoxicity, without excluding outliers. Histopathology and immunohistochemistry were performed in blind.

### Ex-vivo analysis of tumor cryosections

Tumors (n = 8/group) resected at day 24, were sectioned into two similar parts. One has been processed for cryosectioning, and the other has been treated for paraffin embedding. After a fixation step of 3 h in 4% paraformaldehyde, samples have been washed, embedded in OCT and freezed in liquid nitrogen before cryosectioning. Tumor cryosections (9 µm thick) were thawed at room temperature for 15 min, washed with PBS and labelled with Alexa Fluor 488 wheat germ agglutinin (when necessary). Then, after a permeabilization step of 5 min at room temperature (RT) in PBS supplemented withTriton X-100 (0.1%), a blocking step of 1 h as been performed at RT in PBS supplemented with BSA (2%) and goat serum (2%), followed by the labelling with primary antibodies for 2 h at RT. α-SMA antibody (1:400 in blocking buffer; Cell Signalling Technology #19245), Alexa Fluor 488 anti-human IgG antibody (1:400 dilution in blocking buffer; Life Technology #A-11013) or cleaved caspase 3 antibody (1:300 diluition in blocking buffer; Cell signaling Technology D175 #9661) have been respectively used to detect CAF, TZ or apoptosis. α-SMA and anti- cleaved caspase 3 labelled samples were labelled for 2 h at RT with goat Alexa Fluor-546 anti-rabbit secondary antibody (1:400 in blocking buffer; life technology #A-11010) after PBS washing. At the end of the immunocytochemistry reactions, samples were counterstained with DAPI (10 µM, 10 min at RT), washed thrice and mounted with Prolong Gold (Life technology; #P10144). Images of tumor cryosections were acquired at 40 × magnification with a Leica SP8 microscope confocal system using laser excitation lines 405 nm, 488 nm, 535 nm. Resolution of images is of 1,024 × 1,024 or 512 × 512 pixel.

### Immunohistochemistry

Resected D2F2/E2 tumors formalin-fixed and paraffin embedded were sectioned into Three micrometer thick slices (n = 5/group). Samples were processed for CD31 immunohistochemistry as previously described^9^. The number of vessels was counted on 10 fields/sample, field size 1 mm × 1 mm.

### Apoptosis assay

Paraffin-embedded tumor sections (n = 8/group; Three micrometer thick) were processed with the Tumor TACS In Situ Apoptosis Detection kit (Trevigen)^9^ or with Click-iT TUNEL Colorimetric ICH Detection kit (Invitrogen) according to manufacturer’s protocols. Apoptotic nuclei labeled by diaminobenzidine were counted on 10 squares/sample (n = 8/group). Magnification 20 × .

### Flow cytometry

D2F2/E2 tumors surgically removed from treated mice (n = 4/group) were isolated at day 24 and dissociated in single cell suspension by tumor dissociation kit (Miltenyi # 130-096-730) and gentleMACS dissociator (Miltenyi # 130-093-235) following the manufacturer’s instructions. A staining of single cell suspension has been performed with anti-CD45 (clone 30F11, Miltenyi), anti-CD3 (clone REA641, Miltenyi) and anti-CD49b (clone DX5, Miltenyi) antibodies. Data were collected on an Gallios cytometer (Beckman Coulter) and analysed with FlowJo software (TreeStar).

### Histopathological analysis

Liver, kidneys, spleen, heart, brain, gut and lung samples obtained from Balb/C mice (n = 3/group) were fixed in 10% buffered formalin for at least 48 h and embedded in paraffin^9^. Three micrometer sections were cut, stained with hematoxylin and eosin, and examined in a blinded manner^9^.

### Ultrastructural analysis (TEM)

From 4 mice/group, a portion of heart tissue excised during the sacrifice has been fixed in 2.5% glutaraldehyde in 0.1 M phosphate buffer, pH 7.2, for 2 h. After one rinsing with PBS, specimens were post-fixed in 1.5% osmium tetroxide for 2 h, dehydrated by 50, 70, 90, and 100% EtOH, and embedded in epoxy resin (PolyBed 812 Polysciences Inc.). Ultrathin sections were cut with an ultramicrotome (Ultracut E (Reichert-Jung)), stained with uranyl acetate and lead citrate and examined by TEM (Tecnai Spirit, FEI)^9^. For mitochondria quantification at least 10 images/group were taken at 4,200 × magnification^9^. Four mice were used for each experimental condition. Mitochondrial morphometric measurements were performed using ImageJ software on at least 10 images/group acquired at 11.500 × magnification measuring the area of at least 100 mitochondria/sample^9^. The percentage of area occupied by mitochondrial cristae was measured by ImageJ software imposing a threshold value of 118^9^.

### Assessment of kidney and liver functionality

Kidney and liver functionality were assessed as previously described (n = 6/group), using the following kits: QuantiChromTM Urea Assay Kit, QuantiChromTM Creatinine Assay Kit, EnzyChromTM Aspartate Transaminase Assay Kit and EnzyChromTM Alanine Transaminase Assay Kit (BioAssay Systems)^9^.

### Doxorubicin quantification in heart

Heart from treated mice (n = 3–4/group) were processed and quantified by a liquid chromatography tandem mass spectrometry as previously reported^29^. The heart tissue matrix was added to the published method. Sensitivity, specificity, precision, accuracy, recovery and matrix effect of extraction and quantification procedures have been assessed in compliance to the US Food and Drug Administration (FDA) guidelines for bioanalytical methods’ validation. Quantification were performed using Daunorubicine and Doxorubicinol as internal standards on standard curve prepared in heart homogenates . The data are the mean ± SE of samples collected from at least 3 different mice.

### Western blot

D2F2/E2 tumors and hearts from treated mice (n = 3/group) were isolated at day 24 and homogenized in water (10% w/v)^29^. After a lysis step of 30 min at 4 °C in 20 mM Tris HCl pH 7.6, 150 mM NaCl, 1 mM EDTA, 1% Triton X-100, 1% glycerol, 1 mM Na_3_VO_4_, 10 mM NaF, Protease Inhibitor Cocktail, 1 mM PMSF, samples were centrifuged (6,000 × *g* for 10 min at 4 °C) to discard cell debris. Protein content was determined by Bradford assay, BSA has been used to obtain a standard curve. For evaluation of Granzyme B, approximately 30 µg of lysate was loaded on SDS-PAGE (10% acrylamide) and blotted onto PVDF membrane. After a blocking step of 1 h at RT in blocking buffer (TBS supplemented with nonfat dry milk (5%) and Tween 20 (0.1%)), membrane was labelled with anti-Granzyme B antibody (Cell Signalling Technlogy #4275; 1:1,000 in blocking buffer) at 4 °C overnight. Then, a washing step was performed using TBS supplemented with 0.1% Tween 20 and repeated twice, before 1 h at RT incubation with goat Anti-rabbit-HRP secondary antibody in blocking buffer (Abcam, #ab97200; dilution 1:4,000). Finally, the washing step was performed thrice. To evaluate TZ accumulation, approximately 35 µg of protein from each sample were loaded on SDS-PAGE (12% acrylamide). Then, proteins were transferred to the membrane (PVDF; Sigma-Aldrich) and blocked in in blocking buffer (TBS supplemented with nonfat dry milk (5%) and Tween 20 (0.1%))for 1 h. TZ was labelled by overnight incubation at 4 °C with goat anti-human monoclonal antibody conjugated with horseradish peroxidase (Tebu-bio, #GTX26759) at 1:2000 dilution in washing buffer (TBS with 0.1% Tween 20). After a washing step repeated thrice, the ECL star reagent (Euroclone) was used for chemioluminescence emission, which is capture by the Chemidoc System (Biorad).

### Statistical analysis

T-test, one-way ANOVA or Kruskal-Wallis tests performing multiple comparison have been used for statistical analysis, assuming normal distribution. P-values < 0.05 were considered as statistical significant.

### Ethics statements

Investigation has been performed according to national and international guidelines and to Declaration of Helsinki. Moreover, it has been approved by the institutional review board of the University of Milan. A protocol about activity on animals has been approved by the Italian Ministry of Health (Authorization number 520/2017-PR).

## Data availablility

Raw data will be available upon request at the following URL https://doi.org/10.13130/RD_UNIMI/I4YV97.

## Supplementary information

Supplementary file1 (PDF 2105 kb)
