# Supplementary material for: Co-administration of H-ferritin-doxorubicin and Trastuzumab in neoadjuvant setting improves efficacy and prevents cardiotoxicity in HER2 + murine breast cancer model
Source: Sci Rep. 2020 Jul 10;10:11425. doi: 10.1038/s41598-020-68205-w (PMC7351752; doi:10.1038/s41598-020-68205-w)
Supplement: Supplementary file 1 — Supplementary file1 (PDF 2105 kb) [file 41598_2020_68205_MOESM1_ESM.pdf]

Supporting informations

Co-administration of H-ferritin-doxorubicin and Trastuzumab in neoadjuvant setting improves efficacy and prevents cardiotoxicity in HER2+ murine breast cancer model

Andreata F.,[1#§] Bonizzi A.,[1#] Sevieri M.,[1] Truffi M.,[1] Monieri M.,[1,§§] Sitia L.,[1] Silva F.,[1] Sorrentino L.,[1,2]Allevi R.,[1] Zerbi P.,[3] Marchini B.,[3] Longhi E.,[3] Ottria R.,[4] Casati S.,[7] Vanna R.,[5] Morasso C.,[5] Bellini M.,[6] Prosperi D.,[6] Corsi F.,[1,2,5,*] Mazzucchelli S.[1,*]

[1]Nanomedicine Laboratory, Department of Biomedical and Clinical Sciences "Luigi Sacco", Università di Milano, Milan, Italy.

[2] Breast Unit, Istituti Clinici Scientifici Maugeri IRCCS, Pavia, Italy.

[3] Pathology Unit, Department of Biomedical and Clinical Sciences "Luigi Sacco", Università di Milano, Milan, Italy.

[4] Medical Chemistry, Department of Biomedical and Clinical Sciences "Luigi Sacco", Università di Milano, Milan, Italy.

[5] Nanomedicine and Molecular Imaging Laboratory, Istituti Clinici Scientifici Maugeri IRCCS, Pavia, Italy.

[6] NanoBioLab, Department of Biotechnology and Biosciences, Università di Milano -Bicocca, Milan, Italy.

[7] Department of Biomedical, Surgical and Dental Sciences, Sezione di Tossicologia Forense, Università di Milano, Milan, Italy

[#] equally contributed

[§] present address: Division of Immunology, Transplantation and Infectious Diseases, IRCCS San Raffaele Scientific Institute and Vita-Salute San Raffaele University, Milan 20132, Italy.

[§§] present address: Tumor Biology and Vascular targeting Unit, Division of Experimental Oncology, IRCCS San Raffaele Scientific Institute and Vita-Salute San Raffaele University, Milan 20132, Italy.

[*]Corresponding Authors

Serena Mazzucchelli, PhD; phone:+39 0239044050; email: serena.mazzucchelli@unimi.it

Fabio Corsi, Prof., MD; phone: +39 0250319850; email: fabio.corsi@icsmaugeri.it

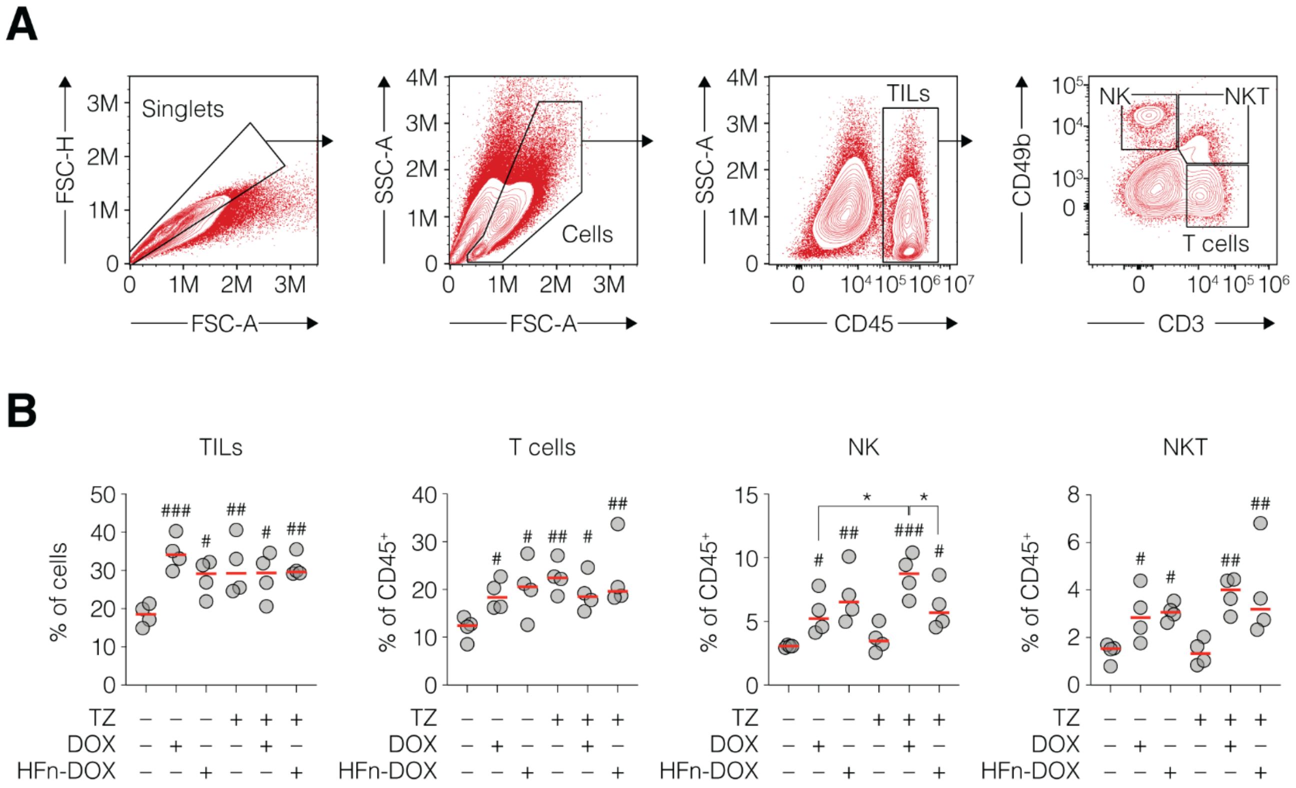

Supplementary Figure 1. Flow-cytometry analysis of tumor infiltrating leukocytes. Tumor samples coming from D2F2/E2 tumor-bearing mice were enzymatically digested in order to obtain a single cell suspension suitable for flow-cytometry. **A**. Gating strategy used to identify total tumor infiltrating leukocytes (defined as $CD45^{+}$ cells), Natural Killer cells (NK, $CD45^{+}/CD3^{-}/CD49b^{+}$), T cells ($CD45^{+}/CD3^{+}/CD49b^{-}$) and NK-T cells ($CD45^{+}/CD3^{+}/CD49b^{+}$). **B**. Frequencies of leukocytes populations among total tumor mass (n=4/group, values are reported as mean showing all values). Statistical significance vs. placebo, # P<0.05, ## P<0.01, ### P<0.005; vs DOX+TZ, * P<0.05 (One-way ANOVA).

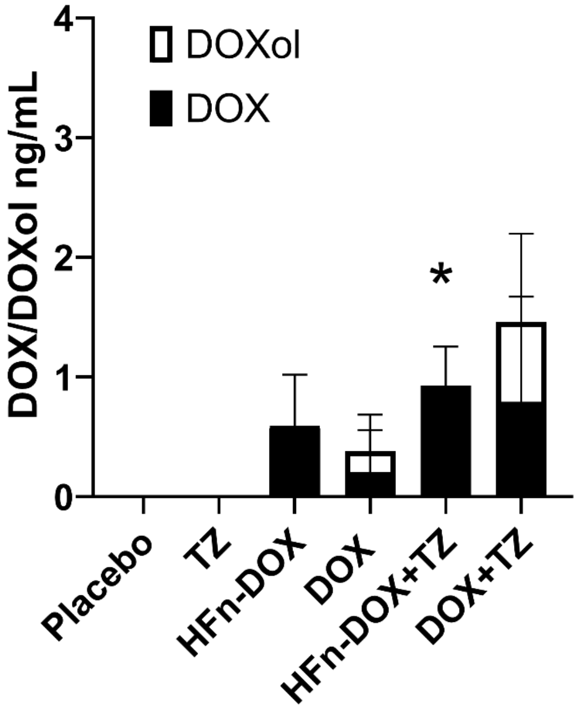

Supplementary Figure 2. DOX quantification in heart tissue. Heart homogenates from D2F2/E2 tumor bearing mice sacrificed at day 25 after the treatment with HFn-DOX or free DOX (1 mg Kg^{-1} of DOX, i.v.) as single agents or in combination with TZ (5 mg/Kg, i. p.) have been processed for DOX and DOXol quantification. DOX and DOXol levels in hearts have been determined following acidified isopropanol extraction from tissue homogenates. Aliquots have been extracted and analyzed by HPLC/MS-MS. Reported values are means of 3-4 samples/group ± s.e. DOX concentration statistical significance vs placebo *$p<0.05$ (One-way ANOVA).

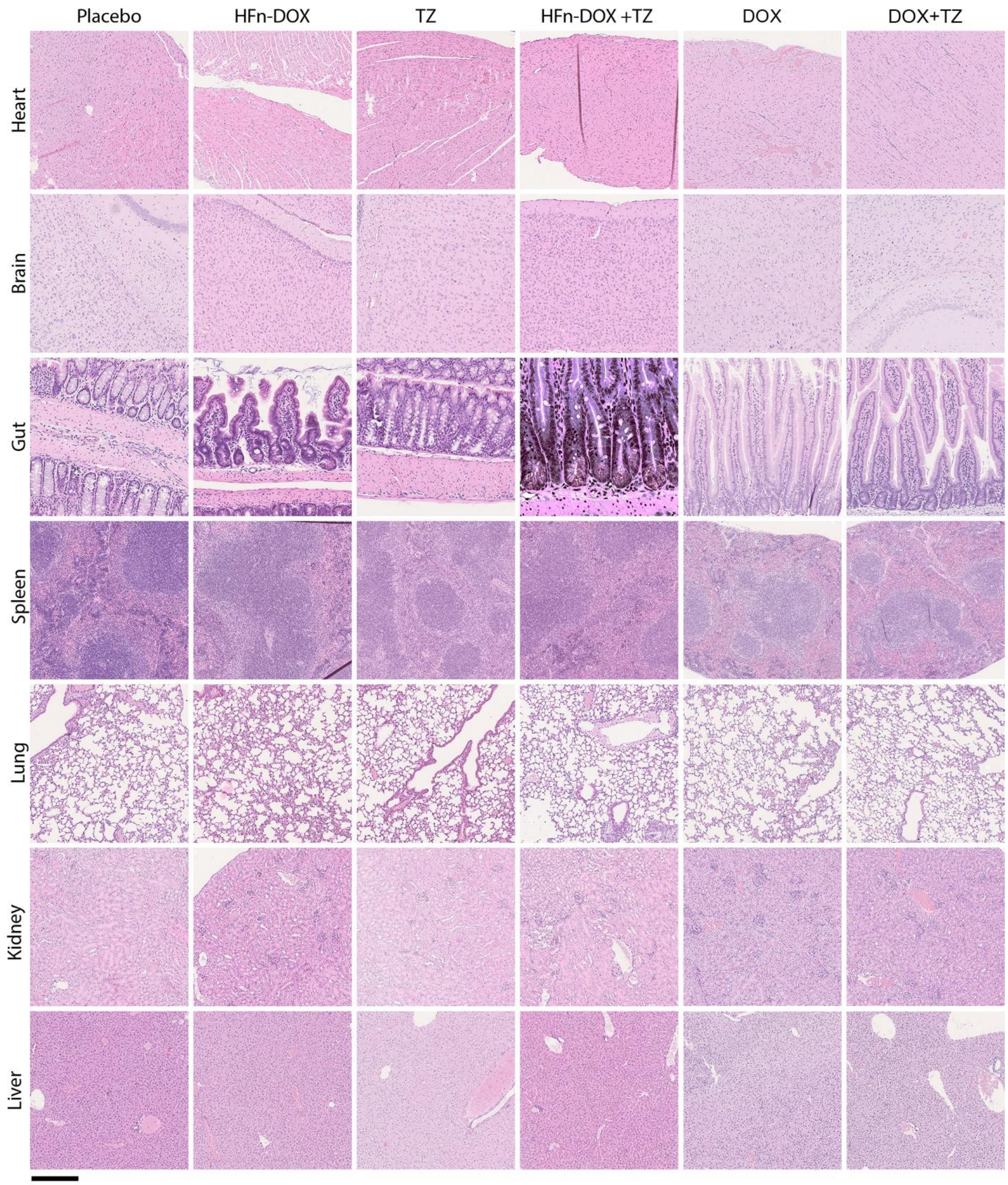

Supplementary Figure 3. Representative images of liver, spleen, kidneys, lungs, heart, gut and brain excised from D2F2/E2 tumor bearing mice after the treatment with placebo, HFn-DOX or DOX (1 mg/Kg of DOX, i.v.), TZ (5 mg/Kg, i. p.) and with the combinations of HFn-DOX+TZ and DOX+TZ. No histological lesions were observed in organs excised from mice differently treated. Hematoxylin-

eosin staining. Magnification 10×. Scale bar = 250 µm. In Gut images magnification is 20× and scale bar = 100 µm.

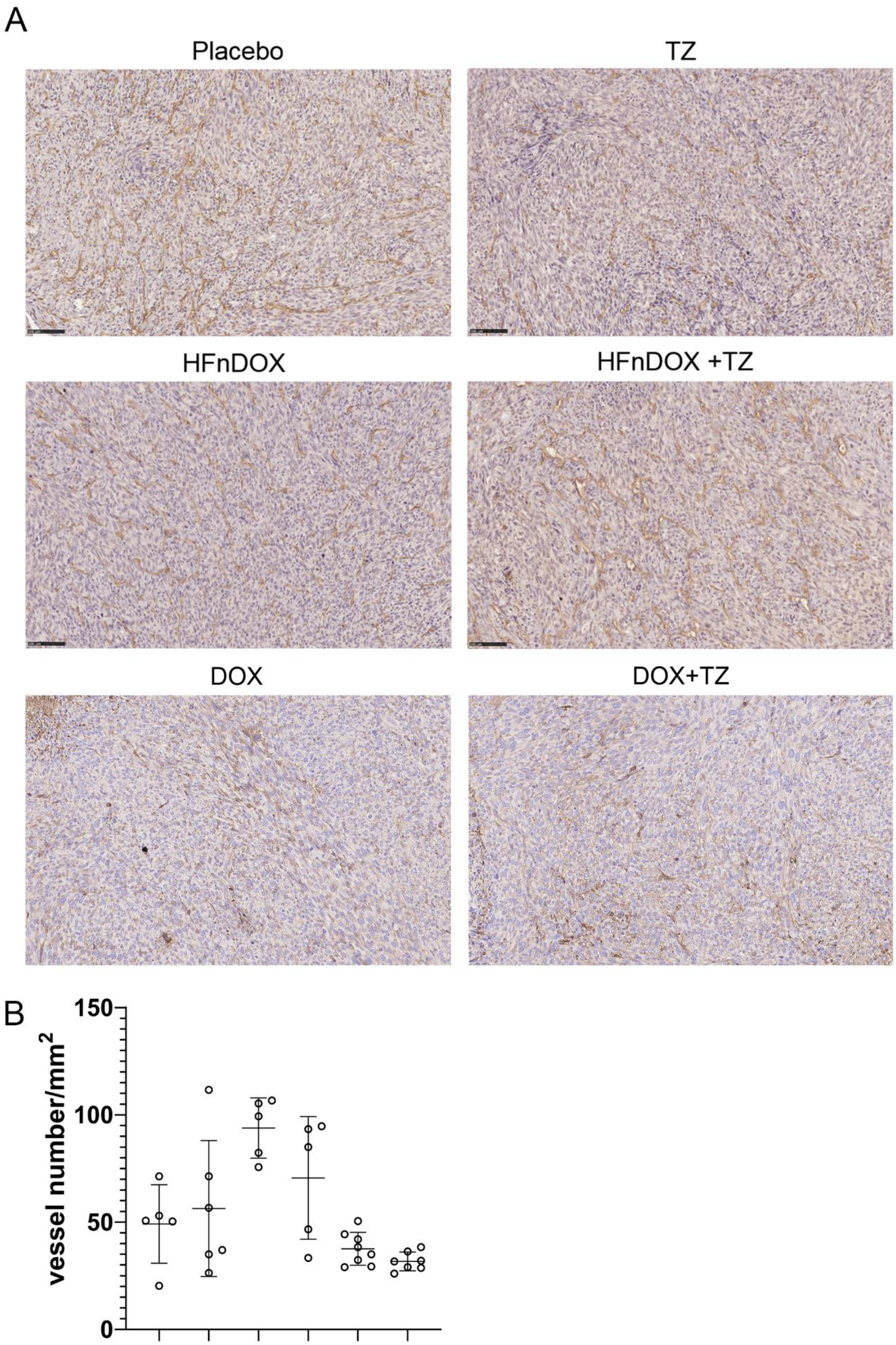
A
Placebo
TZ
HFnDOX
HFnDOX +TZ
DOX
DOX+TZ
B
vessel number/mm2
150
100
50
0
Placebo
TZ
HFn-DOX
HFn-DOX+TZ
DOX
DOX+TZ

Supplementary Figure 4. Effects of HFn-DOX and TZ combination on tumor angiogenesis. **A.** CD31 immunohistochemistry of D2F2/E2 tumors excised at day 24 from Balb/C mice treated with placebo, HFn-DOX or DOX (1 mg/Kg of DOX, i.v.), TZ (5 mg/Kg, i. p.) and with the combinations of HFn-DOX+TZ and DOX+TZ. Magnification 20×. Scale bar 100μm **B.** Quantification of vessel number performed on FFPE tumors excised at day 25 (n = 5/group). Field size 1mm×1mm. Reported values are the mean of vessel number/field/sample ± SE. The count was performed on 3 fields/sample.

Supplementary Table 1. Liver functionality of mice treated with placebo, HFn-DOX, TZ and with HFn-DOX and TZ combination.

		**Mean**	**s.e.**	**P-value**
AST (U L^{-1})	placebo	36,96432854	4,069223503	-
	HFn-DOX	42,91127098	3,239904504	0,194710542
	DOX	33,26080509	3,772816825	0,839418654
	TZ	42,911271	3,2399045	0,27009839
	HFn-DOX and TZ	46,05965228	3,328095365	0,120826831
	DOX+TZ	37,46137881	3,659417082	0,334828747
ALT (U L^{-1})	placebo	13,8867909	5,52982043	-
	HFn-DOX	21,72198623	4,19073638	0,322347023
	DOX	21,15692462	2,513444357	0,245158774
	TZ	22,75644702	4,788758462	0,383323239
	HFn-DOX and TZ	29,83979893	3,95092651	0,001280712
	DOX and TZ	19,53864045	2,30569933	0,400592633

Reference values of healthy female Balb/C mice of 8-10 weeks are 54-298 U mL^{-1} for AST, while 15-84 U mL^{-1} for ALT. P- values have been determined using *t-test.*

Supplementary Table 2. Kidney functionality of mice treated with placebo, HFn-DOX, TZ and with HFn-DOX and TZ combination. P- values have been determined using *t-test.*

		**Mean**	**s.e.**	**P-value**
Urea (mg dL^{-1})	placebo	48,11769006	2,967058199	-
	HFn-DOX	47,60599415	5,743698654	0,944170686
	DOX	45,61048922	4,464399068	0,577723357
	TZ	49,71978558	2,44811507	0,751214903
	HFn-DOX + TZ	50,63048246	5,743767943	0,75174141
	DOX+TZ	37,97348485	4,014829788	0,112684832
Creatinine (mg dL^{-1})	placebo	4,393023203	0,15030935	-
	HFn-DOX	5,280354566	0,303323145	0,010819897
	DOX	3,58958523	0,132275835	0,0143536
	TZ	4,393023203	0,15030935	0,101939479
	HFn-DOX + TZ	4,858104858	0,399429791	0,203110664
	DOX+TZ	2,87559257	0,186051514	0,0016316

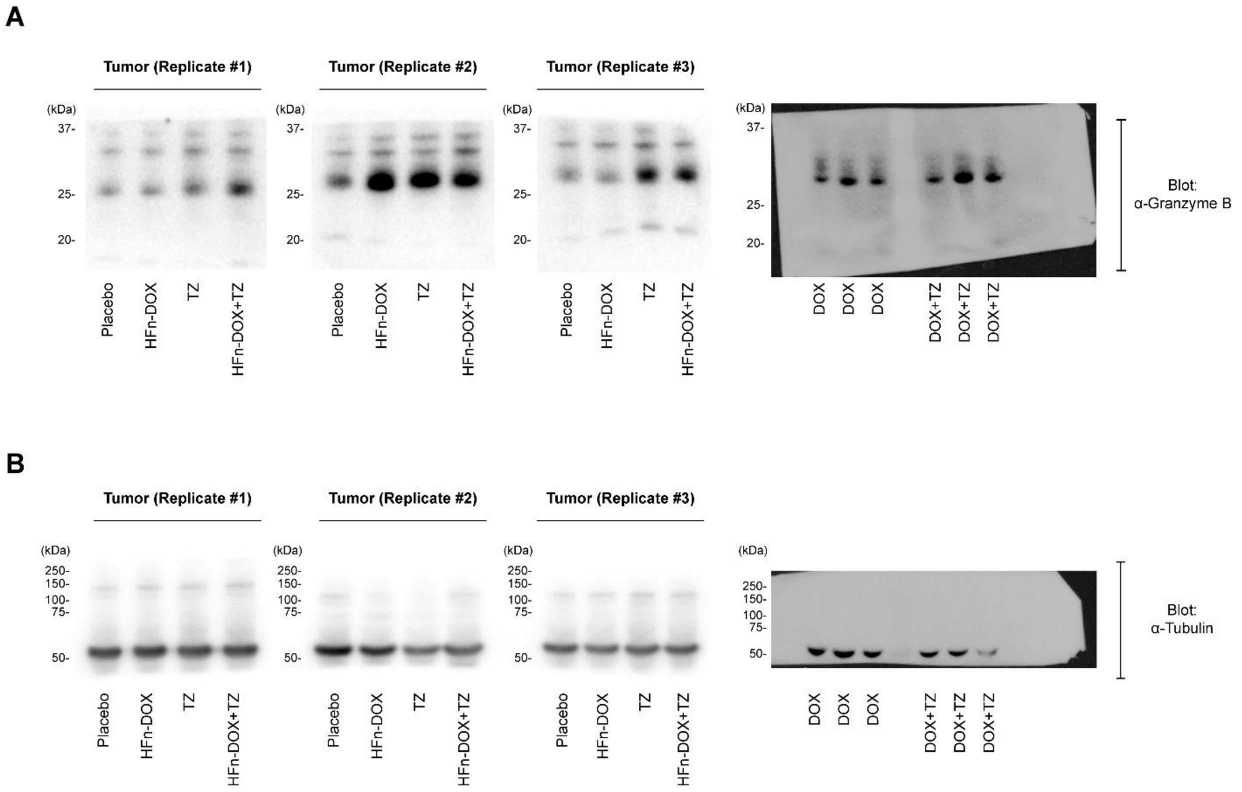

Figure S5. Original Western blots used in figure 1E and 1F. 30 µg of total protein lysates obtained from tumors excised at the end of treatment, as described in the main text, were loaded onto a 10% polyacrylamide. Resolved proteins were blotted onto a PVDF membrane and stained for (**A**) Granzyme B and (**B**) for Tubulin. Three gels have been made and each of them has been loaded with a different replicate of tumors coming from the different groups (Placebo, HFn-DOX, DOX, TZ, HFn-DOX+TZ and DOX+TZ). Those replicates have been used to make the quantification with ImageJ. Tubulin was used as the reference protein in order to not interfere with the expected molecular weight of Granzyme B.

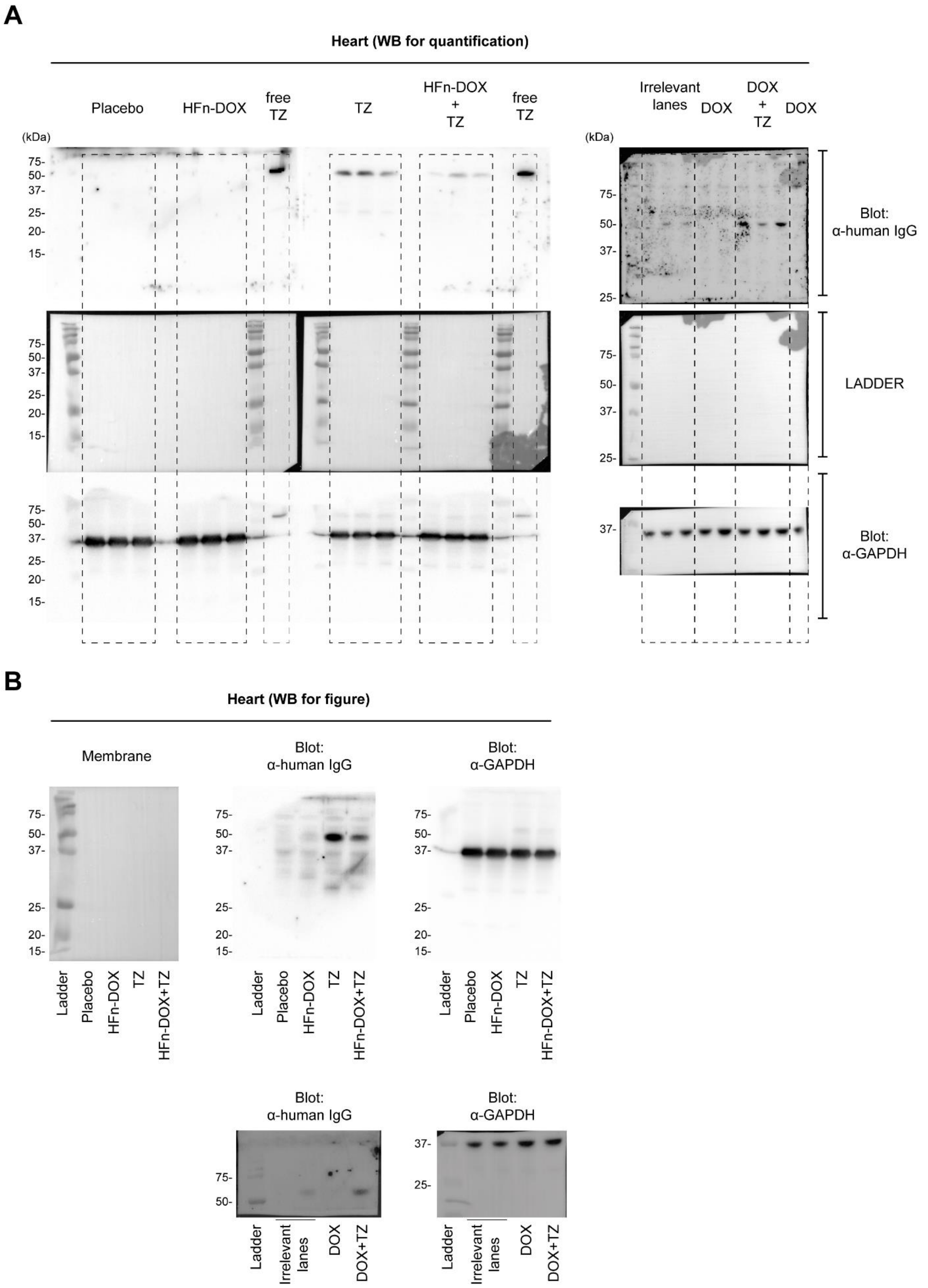
A
Heart (WB for quantification)
Placebo
HFn-DOX
free TZ
TZ
HFn-DOX + TZ
free TZ
Irrelevant lanes
DOX
DOX + TZ
DOX
(kDa)
75-
50-
37-
25-
20-
15-
Blot: α-human IgG
LADDER
Blot: α-GAPDH
B
Heart (WB for figure)
Membrane
Blot: α-human IgG
Blot: α-GAPDH
Ladder
Placebo
HFn-DOX
TZ
HFn-DOX+TZ
Irrelevant lanes
DOX
DOX+TZ

Figure S6 Original Western blots used in figure 3A and 3B. 30 µg of total hearts lysates obtained from mice sacrificed at the end of treatment were loaded onto a 10% polyacrylamide gel. Resolved proteins were blotted onto a PVDF membrane and stained with a goat anti-human IgG-HRP antibody in order to detect intracardiac Trastuzumab. Blots were also stained for GAPDH as a loading control (this reference protein was chosen so as to not interfere with the molecular weight of Trastuzumab). (**A**) Western blots used for the quantification were loaded with three different hearts belonging to the group of this study (Placebo, HFn-DOX, DOX, TZ, HFn-DOX+TZ and DOX+TZ); 20 ng of purified Trastuzumab were also included in these gels as an internal positive control for the staining. (**B**) One heart sample per group was loaded on a different gel and stained for human-IgG and GAPDH.

A

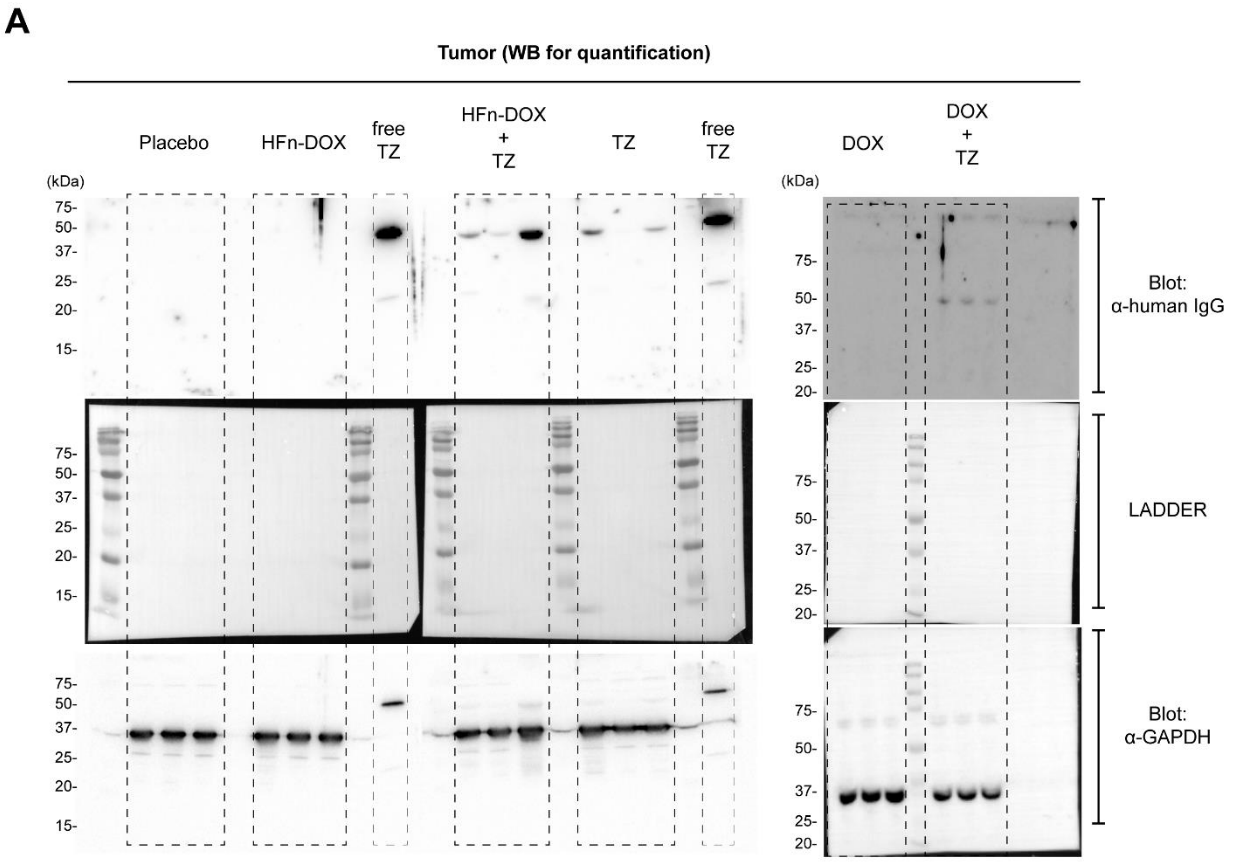
Tumor (WB for quantification)
Placebo
HFn-DOX
free TZ
HFn-DOX + TZ
TZ
free TZ
DOX
DOX + TZ
(kDa)
75-
50-
37-
25-
20-
15-
Blot: α-human IgG
LADDER
Blot: α-GAPDH

B

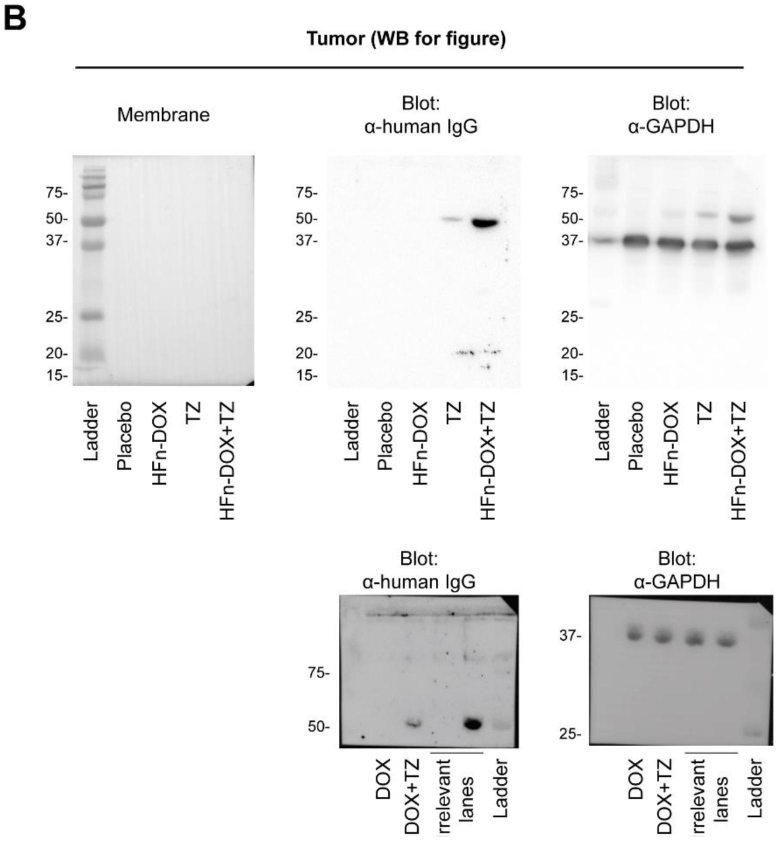
Tumor (WB for figure)
Membrane
Blot: α-human IgG
Blot: α-GAPDH
75-
50-
37-
25-
20-
15-
Ladder
Placebo
HFn-DOX
TZ
HFn-DOX+TZ
DOX
DOX+TZ
rrelevant lanes
Ladder

Figure S7 Original Western blots used in figure 3C and 3D. 30 µg of total protein lysates obtained from tumors excised at the end of treatment, as described in the main text, were loaded onto a 10% polyacrylamide gel. Resolved proteins were blotted onto a PVDF membrane and stained with a goat anti-human IgG-HRP antibody in order to detect intratumoral Trastuzumab. Blots were also stained for GAPDH as a loading control (this reference protein was choosen so as to not interfere with the molecular weight of Trastuzumab). (**A**) Western blots used for the quantification were loaded with three different tumors belonging to the group of this study (Placebo, DOX, HFn-DOX, TZ, DOX+TZ and HFn-DOX+TZ); 20 ng of purified Trastuzumab were also included in these gels as an internal positive control for the staining. (**B**) One tumor sample per group was loaded on a different gel and stained for human-IgG and GAPDH.
